# Laminar air flow reduces particle load in TKA—even outside the LAF panel: a prospective, randomized cohort study

**DOI:** 10.1007/s00167-020-06344-3

**Published:** 2020-11-09

**Authors:** Stephanie Kirschbaum, Hagen Hommel, Peggy Strache, Roland Horn, Roman Falk, Carsten Perka

**Affiliations:** 1 Centre for Musculoskeletal Surgery, Charité - University Hospital Berlin, Charitéplatz 1, 10117 Berlin, Germany; 2 Clinic for Orthopaedics, Sports Medicine and Rehabilitation, KH-MOL GmBH, Sonnenburger Weg 3, 16269 Wriezen, Germany; 3 Academic Teaching Hospital of the Medical School of Brandenburg Theodor Fontane, Fehrbelliner Strase 38, 16816 Neuruppin, Germany

**Keywords:** Particle sizes, Particulate matter, Airborne particulate matter, Surgical site infection, Laminar air flow areas, Total knee arthroplasty, Total knee replacement

## Abstract

**Purpose:**

Released particles are a major risk of airborne contamination during surgery. The present prospective study investigated the quantitative and qualitative particle load in the operating room (OR) depending on location, time of surgery and use of laminar air flow (LAF) system.

**Methods:**

The particle load/m^3^ was measured during the implantation of 12 total knee arthroplasties (6 × LAF, 6 × Non-LAF) by using the Met One HHPC 6 + device (Beckmann Coulter GmbH, Germany). Measurement was based on the absorption and scattering of (laser) light by particles and was performed at three different time-points [empty OR, setting up, ongoing operation) at 3 fixed measurement points [OR table (central LAF area), anaesthesia tower (marginal LAF area), surgical image amplifier (outside LAF area)].

**Results:**

Independent of time and location, all measurements showed a significantly higher particle load in the Non-LAF group (*p* < 0.01). With ongoing surgical procedure both groups showed increasing particle load. While there was a major increase of fine particles (size < 1 µm) with advancing activity in the LAF group, the Non-LAF group showed higher particle gain with increasing particle size. The lowest particle load in the LAF group was measured at the operating column, increasing with greater distance from the operating table. The Non-LAF group presented a significantly higher particle load than the LAF group at all locations.

**Conclusion:**

The use of a LAF system significantly reduces the particle load and therefore potential bacterial contamination regardless of the time or place of measurement and therefore seems to be a useful tool for infection prevention. As LAF leads to a significant decrease of respirable particles, it appears to be a protective factor for the health of the surgical team regardless of its use in infection prevention.

**Level of evidence:**

I.

## Introduction

Causes of periprosthetic infections are mostly either a haematogenic spread or an intraoperative contamination [1]. Intraoperative contamination can occur per continuitatem as well as airborne [2–6]. Airborne contamination is linked to the presence of suspended particles [7, 8]. Approximately 5–10% of such particles carry bacteria, allowing bacteria sedimentation and contamination of the operating area or instrument table [7]. As a consequence, particle load can be used as a parameter for risk of infection [7–9].

Various authors demonstrated that the use of laminar air flow (LAF) systems resulted in a reduced intraoperative bacteria sedimentation [2, 5, 10–12]. It is therefore surprising that current literature didn’t find a reduced infection rate when using LAF [13–16]. However, recommendations of existing reviews or meta-analyses examining the use of LAF systems in reduction of surgical site infection (SSI) are usually based on inhomogeneous studies with different types and sizes of LAF systems [13, 14, 17, 18], where there may also be a lack of standardization of possible cofounders (antibiotic prophylaxis, patient related risk factors). Another factor which is often discussed but has not yet been thoroughly examined, is the turbulent air flow occurring at the margin of the LAF panel or even inside the LAF area due to obstacles. Only few studies compare concentration of suspended particles depending on position inside the LAF area or examines particles as potential bacteria carriers in the operative arthroplasty setting [11, 12, 19]. No study currently examines the quantitative and qualitative particle load or its distribution during total knee arthroplasty (TKA) in comparison with or without working of LAF system. The aim of the present study is therefore to evaluate the quantitative and qualitative particle load in the operating room depending on the measurement location (inside LAF area, margin of LAF area and outside LAF panel), the time of surgery and the use of a LAF system. It was hypothesized that the LAF System is able to reduce particle load at any time of surgical procedure. It was furthermore hypothesized that outside the working LAF area particle load increases due to turbulent air flow.

## Methods

This prospective cohort study was approved by the local ethics committee (AS58(bB)/2017). All the patients provided informed consent to be involved in the study.

### Study design

The air particle concentration was measured during the implantation of 12 TKA. All patients were informed about the aims and the design of the study and agreed to participate. The patients were randomly allocated to LAF or Non-LAF groups. Only the study nurse performing the measurements was informed about the randomization. The surgical team was blinded concerning the LAF function. Six TKA were implanted while using a LAF system (LAF group), 6 TKA without the use of LAF system (Non-LAF group). One measurement failed due to technical problems and therefore was not included in the data analysis. A new case was therefore included. In order to avoid cofounders due to architecture or previous operations, recommendations of Edmiston et al. were followed [20]: Every TKA evaluated in this study was performed as first position in the same operation room (OR).

The measuring method of the Met One HHPC 6 + device (Beckmann Coulter GmbH, Germany) is based on the absorption and scattering of (laser) light by particles. Photodiodes detect these effects and convert them into electrical signals, which are counted accordingly by the device. The measuring device can detect particles with a diameter of 0.3–10 μm. Each measurement took 120 s and examined an air volume of 5.66 l.

Measurement was performed at three different time points during the ongoing operation. First, the particle load was referenced before the beginning of the operation day without any persons in the OR. The second measurement was performed after preparing the surgical setting but before the patient entered the OR. The third measurement was performed after exposure of the knee joint using the electrocautery ("SafeAir Smoke Evacuator", Stryker) but before saw cuts were made.

**Fig. 1:**
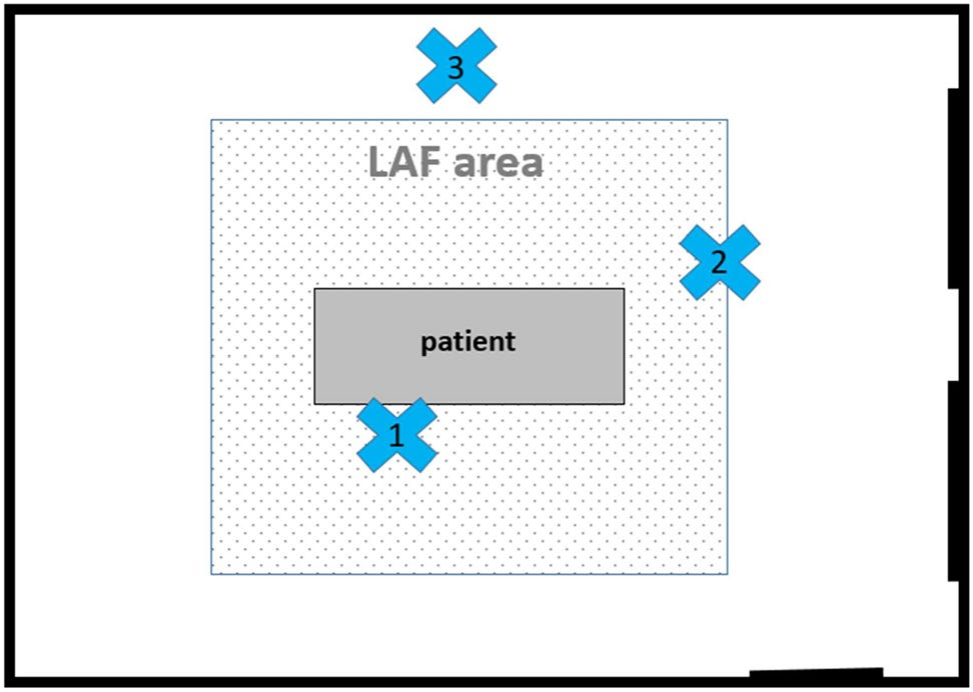
Measurement points in the operating room, 1—next to the column, centrally in the LAF area, 2—anaesthesia tower, margin of LAF area, 3—surgical image amplifier, outside the LAF area

The particle load each time was measured at three fixed positions within the operating theatre (Fig. 1). Position 1 was located centrally under the laminar flow system, directly next to the operating column. Position 2 was located at a defined point of the anaesthesia device. This is located marginal in the LAF area. Position 3 was defined as a control point outside the LAF area near the surgical image amplifier.

### Microbiological examination

To test for airborne contamination due to particle load, a single swab (Fa. COPAN – FLOQ Swabs) of the electrocauteries “SafeAir Smoke Evacuator” filter was taken after each operation (*n* = 14). All microbiological samples were cultured on blood agar plates (37 °C, 48 h) in a standard fashion and interpreted by a consultant microbiologist.

### LAF system

The panel of the LAF system (Admeco, Hochdorf, Switzerland) measured 3.2 × 3.2 m. Air volume flow was 11,400m^3^/h, the vertical flow velocity reached 0.22 m/s. The exhaust air extraction was 50% near the floor and 50% by the ceiling device. The complete surgical team as well as all instrument tables were placed beneath the LAF area.

### Door opening and surgical team

In order to minimize cofounders such as increased particle load due to door opening and number of persons in the OR, those risk factors were standardized [19, 21]. Door opening was reduced to a minimum: study nurse entered an empty OR, 2 scrub nurses entered OR to prepare surgical setting, patient and anaesthesiology team (2 persons) entered OR and surgical team entered OR.

Once the patient entered the room, there were seven persons in the OR. All employees were dressed in cotton-blended clothing in accordance with the applicable hygiene regulations and were equipped with surgical hoods and masks. The core team (2 surgeons, 1 instrumental surgical assistant) also wore protective goggles, sterile gloves and disposable gowns from Berendsen Chirutex.

### Statistical evaluation

Based on the results of Sossai et al. [12], the sample size estimation of the current study was performed using the program G*Power (Version 3.1, 2014) with an effect strength of 2.33, *α* = 0.05 and a power of 95%. First, a general comparison of qualitative and quantitative particle load between LAF and Non-LAF groups, independent from measurement time and location, was performed. Furthermore, particle load was analysed depending of measurement time and measurement location. Descriptive statistics including means, standard deviation, and minimum and maximum values of continuous variables within the groups were calculated using the Friedman Test. The mean values between LAF and Non-LAF were compared using the Mann–Whitney Test (MWU). A *p*-value below 0.05 was considered significant.

## Results

### General comparison of LAF and Non-LAF measurements

Table 1 shows a comparison of all particle load measurements between activated and non-activated LAF systems, independent of time and location.

**Table 1:** Comparison of the particle load of all measurements between LAF and NON-LAF group, independent of time and place of measurement

	*n*	0.3 µm	0.5 µm	1 µm	2 µm	5 µm	10 µm
LAF	54	6,477,729 ± 32,922,983	570,462 ± 2,661,664	56,998 ± 233,118	12,636 ± 46,478	972 ± 2546	331 ± 832
Non-LAF	54	33,866,549 ± 43,453,521	3,036,219 ± 2,637,291	199,221 ± 231,718	47,707 ± 64,023	7885 ± 9491	3265 ± 4178
Factor		5.2	5.3	3.5	3.8	8.1	9.7
*p* (Mann–Whitney-Test)		*p* < 0.01	*p* < 0.01	*p* < 0.01	*p* < 0.01	*p* < 0.01	*p* < 0.01

*LAF* laminar air flow

### Particle load and measurement time

With and without LAF system, a significant increase in particle load was observed with increasing activity in the operating room (Table 2). The dedicated comparison showed at each time-point a significant difference in favour of the LAF group (MWU, *p* < 0.01 each).

**Table 2:** Particle load with and without LAF depending on time of measurement

	*n*	0.3 µm	0.5 µm	1 µm	2 µm	5 µm	10 µm
LAF							
Empty OR	18	20,632 ± 49,303	2876 ± 6270	452 ± 825	236 ± 473	88 ± 184	59 ± 182
Setting prepared	18	11,406 ± 16,111	3573 ± 3822	1737 ± 2194	1198 ± 1761	560 ± 955	265 ± 786
Ongoing operation	18	19,401,148 ± 55 803,469	1,704,937 ± 4,476,903	168,806 ± 386,736	36,474 ± 76,345	2267 ± 4059	668 ± 1 143
Factor		940	592	373	154	25.7	11.3
* p* (Friedman)		*p* < 0.01	*p* < 0.01	*p* < 0.01	*p* < 0.01	*p* = 0.174	*p* = 0.057
Non -LAF							
Empty OR	18	17,853,868 ± 12,096,453	1,553,190 ± 1,021,798	64,291 ± 30,292	7636 ± 2707	825 ± 581	353 ± 321
Setting prepared	18	26,792,344 ± 11,271,949	2,778,190 ± 1,515,588	167,070 ± 83,685	39,988 ± 11,521	8157 ± 2467	3455 ± 1475
Ongoing operation	18	56,953,435 ± 68,735,379	4,777,277 ± 3,568,511	366,304 ± 330,977	95,495 ± 91,967	14,674 ± 13,143	5987 ± 5940
Factor		3.2	3.1	5.7	12.5	17.8	16.9
* p* (Friedman)		*p* < 0.01	*p* < 0.01	*p* < 0.01	*p* < 0.01	*p* < 0.01	*p* < 0.01

### Particle load and measurement place

There was an increase of the particle concentration in the LAF group with increasing distance to the operating column (Table 3).

**Table 3:** Particle load with and without LAF application depending on the measurement place

	*n*	0.3 µm	0.5 µm	1 µm	2 µm	5 µm	10 µm
LAF							
Operation column (central LAF area)	18	8068 ± 10,197	3298 ± 4464	1845 ± 2991	1207 ± 2229	452 ± 937	196 ± 457
Anaesthesia device (marginal LAF area)	18	3,991,490 ± 16,915,423	264,144 ± 1,111,856	42,138 ± 174,027	12,466 ± 49,523	1217 ± 3747	442 ± 1303
Surgical image amplifier (outside LAF area)	18	15,433 628 ± 54,381,711	1,443, 944 ± 4,427,504	127,012 ± 361,229	24,234 ± 63,219	1247 ± 2202	354 ± 469
Factor		1913	438	69	20	2.8	1.8
* p* (Friedman)		*p* < 0.01	*p* < 0.01	*p* < 0.01	*p* < 0.01	*p* = 0.174	*p* = 0.57
Non- LAF							
Operation column (central LAF area)	18	28,558,716 ± 18,156,222	2,853,808 ± 1,844,888	185,061 ± 128,992	47,753 ± 46,313	9089 ± 9160	3828 ± 3893
Anaesthesia device (marginal LAF area)	18	25,956,930 ± 14,670,502	2,735,748 ± 1,774,967	170,770 ± 116,376	43,914 ± 48,841	8147 ± 11,342	3534 ± 5596
Surgical image amplifier (outside LAF area)	18	47 084 000 ± 71 139 621	3 519 101 ± 3 840 879	241 834 ± 366 363	51,453 ± 90,657	6419 ± 8026	2434 ± 2612
Factor		1.6	1.2	1.3	1.1	0.7	0.6
* p* (Friedman)		*p* < 0.01	*p* < 0.01	*p* < 0.01	*p* < 0.01	*p* < 0.01	*p* < 0.01

*LAF* laminar air flow

A comparison of the LAF group and the Non-LAF group showed a significantly reduced particle load, independent of particle size and measurement location, when using LAF (MWU, each *p* < 0.001).

### Microbiological examination

None of the microbiological cultures showed bacteria growth after 48 h of incubation.

## Discussion

Up to now there has been a controversial discussion concerning the effect of LAF on the infection rate in arthroplasty surgery due to inhomogeneous data as well as the lack of prospective well-designed cohort studies. This is the first prospective, randomized cohort study evaluating the influence of LAF on quantitative and qualitative particle load during surgical procedure at several locations of measurement. Although former studies already demonstrated efficacy of the LAF system in reducing overall particle load [11, 12], the current study is the first one able to show that a LAF system using a LAF ceiling device significantly reduces the particle load during the entire surgical procedure at any location—even the one lying outside LAF panel. Those findings were independent from the particle size, confirming our hypothesis.

The current study demonstrated that, independent from the use of LAF device, the particle load increases with ongoing activity and number of persons in the OR. The latter confirmed the finding of Rezapoor et al. The authors demonstrated a decrease in particle density per person from 211.19 to 18.19 particles/ft^3^ (*p* < 0.001) when using a LAF system but failed to evaluate the influence of people activity during surgical procedure [19]. However, the current study showed significant reduction of particle load at any time when using a LAF system. Interestingly, while the LAF group showed the highest increase in small particles during the operating procedure, the Non-LAF group showed the highest increase in bigger particles. So, the LAF system seems more effective in reducing the quantity of bigger particles. As ongoing activity in the OR is proven to increase particle load and therefore increase the number of potential bacteria carriers, it seems reasonable to outsource the patient’s preparation (intubation, shaving, pre-cleaning, positioning) as far as possible from the OR itself in order to reduce particle load and potential bacterial contamination.

In the present study, the LAF group showed an increasing particle load with increasing distance from the operation column by a factor 1.8–1900 depending on particle size. This is not surprising as with increasing distance, decreasing efficacy of the LAF ceiling device can be assumed. Unlike Nilsson et al. suggested [11], the current study was not able to find an increased particle load at the marginal LAF area due to turbulent air flow. On the contrary, this study is the first able to show that LAF system reduces particle load even outside the LAF panel itself. The Non-LAF group showed also a significant increase in particle load (≤ 2 µm) at the operation column compared to the outer OR area, but only by the factor 1.1–1.6. Surprisingly there was no increase within the deactivated LAF area. It remains to be assumed that the LAF ceiling field influences the airflow, and thus the distribution of particles in the operating theatre, even when the LAF is switched off. However, the highest particle load in the LAF group was always less than the lowest particle load in the Non-LAF group at any measurement location, confirming the use of the LAF system in reducing the particle load and therefore the risk of acrogenic bacterial contamination [9].

By now, the possible health-damaging effect of the particle load on the surgical team has received little attention. The effect and damage mechanism depends on surface charge and particle size [22]. The present study showed that the main particle load is made up from alveolar particles < 1 µm, and increases significantly with the beginning of the operation. Here, too, the use of the LAF system achieves a reduction of the load by a factor of 2.1–2.9. Particle sizes up to 2.5 µm are considered respirable while ultrafine particles < 0.1 µm can even enter vessels [23]. Unfortunately, particles smaller than 5 µm cannot be filtered by common surgical masks [24]. A positive surface charge of those ultrafine particles induces activation of the complement system and can trigger thromboembolic events after pulmonary exposure [25, 26]. Furthermore, it was demonstrated that particles are able to carry bacteria as well as viral fragments [27, 28]. Especially during the Corona pandemic, the safety of the OR team by reduction of potential risk factors should be of major interest. Especially as TKA surgery releases high levels of particles and aerosols by the use of saws, drills, electrocautery and Jet Lavage system [29]. Therefore reducing particle load by means of a LAF system might represent an additional safety factor for the surgical team. On the other hand, there are several studies discussing that in the case of Covid-19, operations should take place within negative instead of positive pressure to reduce the risk of disseminating the virus beyond the OR [30–32]. However, reducing particle and therefore virus load requires a high frequency of air changes (25 per h) and the use of a high-efficiency particulate air filter within the OR [31]. Besides the technical setting, correct protective equipment as well as OR team discipline is needed to avoid the spread of Covid-19 beyond the OR [29, 30].

There are some limitations of the current study. First, it must be mentioned that only a smear test, and no evaluation of the CFU/m^3^ or incubation of sedimentation plates, was carried out. Furthermore, there were only 48 h of incubation of the microbiological findings. Slowly growing bacteria such as cutibacterium acnes cannot be detected [33]. Nevertheless, various authors demonstrated that the use of LAF systems resulted in a reduced intraoperative bacteria sedimentation [2, 5, 10–12]. Erichsen et al. showed that LAF systems resulted in a reduction of 89% of colony forming units in comparison with the displacement system [34]. Taken together, LAF reduces the risk of bacterial sedimentation due to effective reduction of particles as bacteria carriers. A third limitation is that here was no examination concerning fungal findings. The lack of microbiological findings in the current study should therefore been viewed critically. Another limitation is the lack measurements carried out at specific time-intervals (rather than at given surgical steps). Although following highly standardized procedures, resulting in similar times of preparation and surgery, a certain bias due to time range cannot be excluded. On the other hand, measuring particle load after a fixed time might result in a bias too, as this results in the comparing of different stages of activity. Furthermore, the current study showed a much higher particle load/m^3^ compared to those mentioned above. Due to a lack of specification regarding model and function of the particle counters used, only a different function can be assumed [2, 5, 10, 11].

A clear distinction must be made between particle load, bacterial contamination and later surgical site infection, as a reduced infection rate using LAF systems could not be confirmed in recent studies [13, 14, 18]. However, as the parameter “infection rate” is strongly dependant on chosen criteria and follow-up time, particle load (as potential bacteria carrier) like used in the current study seems the more reliable parameter in evaluating aerogenic infection risk [7, 9]. Regardless of the infection rate, the LAF system appears to be a protective factor regarding the health burden of the surgical team, as a significant reduction of respirable particles can be achieved.

## Conclusion

The use of a LAF system significantly reduces the particle load and therefore the risk of bacterial contamination regardless of the time or place of measurement and therefore seems to be useful tool for infection prevention. The current study is the first one demonstrating that the LAF system is not only able to reduce general particle load during TKA surgery but is also able to reduce particle load outside the LAF panel. Furthermore, this study was the first one demonstrating that especially respirable particles (< 2.5 µm), which cannot be filtered by the surgical mask, show a major increase in concentration with the beginning of surgery. As the use of LAF leads to a significant decrease of those respirable particles it appears to be a protective factor for the health of the surgical team, regardless of use in infection prevention.

## Abbreviations

OR: Operating room
LAF: Laminar air flow
PJI: Periprosthetic joint infection
SSI: Surgical site infection
TKA: Total knee arthroplasty
CFU: Colony forming units
MWU: Mann–Whitney test
